# From Sunlight to Signaling: Evolutionary Integration of Vitamin D and Sterol Metabolism

**DOI:** 10.3390/metabo16010074

**Published:** 2026-01-14

**Authors:** Marianna Raczyk, Carsten Carlberg

**Affiliations:** InLife Institute of Animal Reproduction and Food Research, Polish Academy of Sciences, 10-683 Olsztyn, Poland; m.raczyk@pan.olsztyn.pl

**Keywords:** vitamin D, sterols, evolution, vitamin D metabolism, VDR, LXR, nutrigenomics

## Abstract

Background/Objectives: This review integrates evolutionary, metabolic, genetic, and nutritional perspectives to explain how sterol-derived vitamin D pathways shape human physiology and inter-individual variability in vitamin D status. Methods: The literature on sterol and vitamin D metabolism across animals, plants, fungi, and algae was synthesized with data from metabolomics databases, genome-wide association studies, RNA-seq resources (including GTEx), structural biology, and functional genomics. Results: Vitamin D_2_ and vitamin D_3_ likely emerged early in evolution as non-enzymatic photochemical sterol derivatives and were later co-opted into a tightly regulated endocrine system in vertebrates. In humans, cytochrome P450 enzymes coordinate vitamin D activation and degradation and intersect with oxysterol production, thereby linking vitamin D signaling to cholesterol and bile acid metabolism. Tissue-specific gene expression and regulatory genetic variants, particularly in the genes *DHCR7*, *CYP2R1*, *CYP27B1*, and *CYP27A1*, contribute to population-level differences in vitamin D status and metabolic outcomes. Structural analyses reveal selective, high-affinity binding of 1,25-dihydroxyvitamin D_3_ to VDR, contrasted with broader, lower-affinity ligand recognition by LXRs. Dietary patterns modulate nuclear receptor signaling through distinct yet convergent ligand sources, including cholesterol-derived oxysterols, oxidized phytosterols, and vitamin D_2_ versus vitamin D_3_. Conclusions: Sterol and vitamin D metabolism constitute an evolutionarily conserved, adaptable network shaped by UV exposure, enzymatic control, genetic variation, and diet. This framework explains inter-individual variability in vitamin D biology and illustrates how evolutionary and dietary modulation of sterol-derived ligands confers functional flexibility to nuclear receptor signaling in human health.

## 1. Introduction

**Figure 1:**
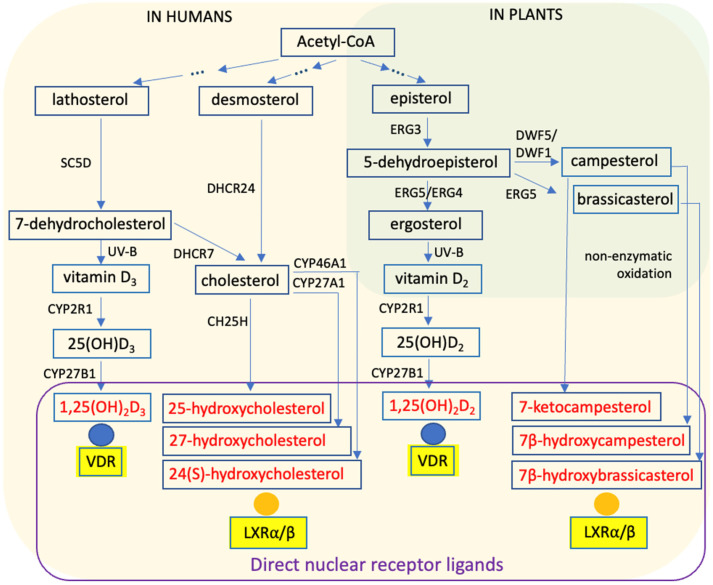
Principles of Steroid Biosynthesis. Steroid biosynthesis pathways in humans (beige) and plants (green) are shown based on the KEGG database (https://www.kegg.jp, accessed on 6 January 2026), with a particular emphasis on metabolites that function as ligands for vitamin D receptor (VDR) and liver X receptors (LXRs). The diagram highlights conserved and lineage-specific enzymatic steps involved in sterol and vitamin D metabolism. SC5D, sterol C5-desaturase; DHCR24, 24-dehydrocholesterol reductase; DHCR7, 7-dehydrocholesterol reductase; CH25H, cholesterol 25-hydroxylase; CYP2R1, cytochrome P450 family 2 subfamily R member 1; ERG3, C5 sterol desaturase; ERG4, C24(28) sterol reductase; ERG5, C22 sterol desaturase; DWF1, sterol side-chain reductase 1; DWF5, Δ7-sterol C5-desaturase.

From sunlight to supplements, vitamin D biology is shaped by evolution, environment, and metabolism. Vitamin D_3_ is a secosteroid produced from the cholesterol precursor 7-dehydrocholesterol upon exposure to solar ultraviolet B (UV-B) radiation (290–320 nm) [1] (Figure 1). In early organisms, vitamin D likely arose as a byproduct of mechanisms protecting against UV radiation. Accordingly, most cholesterol-producing organisms retain the capacity for vitamin D_3_ synthesis upon UV exposure [2]. Consequently, endogenous cutaneous synthesis was, for most of human evolutionary history, the primary source of vitamin D.

Sterol metabolism intersects with vitamin D biology at 7-dehydrocholesterol, a key intermediate in cholesterol biosynthesis [3] (Figure 1). Cholesterol is indispensable for animal physiology, contributing to membrane integrity and fluidity, lipid raft formation, and serving as a precursor for steroid hormones and bile acids [4]. Analogous sterols in other eukaryotes, such as ergosterol in fungi and phytosterols (e.g., campesterol and brassicasterol) in plants, fulfill similar structural functions in membranes and modulate signal transduction and protein localization [5].

In vertebrates, an endocrine vitamin D system emerged approximately 550 million years ago [6,7,8], involving vitamin D-specific metabolic enzymes, binding proteins, and a high-affinity nuclear receptor. The enzyme CYP2R1 converts vitamin D_3_ into 25-hydroxyvitamin D_3_ (25(OH)D_3_), which is subsequently hydroxylated by CYP27B1 to form the hormonally active metabolite 1α,25-dihydroxyvitamin D_3_ (1,25(OH)_2_D_3_) (Figure 1). Acting as an endocrine hormone, 1,25(OH)_2_D_3_ regulates gene expression through the ligand-activated transcription factor VDR [9].

Plants and fungi also produce vitamin D. However, because they primarily contain ergosterol rather than 7-dehydrocholesterol, UV-B irradiation leads to the formation of vitamin D_2_, which differs from vitamin D_3_ in the structure of its side chain [10] (Figure 1). Comparative analyses of vitamin D_2_ and vitamin D_3_ biology reveal both conserved principles of sterol metabolism and lineage-specific adaptations [11,12]. Moreover, genetic polymorphisms shaped by geography and historical UV exposure influence vitamin D synthesis, transport, and signaling, contributing to population-level variability in physiological responses [13]. Contemporary sources of vitamin D, including diet, fortified foods, supplements, and environmental sunlight, interact with these inherited determinants, generating a complex landscape of metabolic and transcriptional outcomes [14,15].

In this review, vitamin D signaling is considered not as an isolated endocrine pathway, but as a specialized branch of a broader sterol–oxysterol network shaped by photochemistry, enzymatic control, and regulatory genetic variation. By integrating evolutionary, metabolic, genetic, and structural perspectives, we aim to clarify why vitamin D metabolism exhibits such pronounced inter-individual variability and how this variability is mechanistically embedded within sterol biology.

## 2. Vitamin D Metabolism: Evolutionary Origins and Ecological Distribution

Vitamin D_2_ and vitamin D_3_ likely originated early in eukaryotic evolution, approximately 1.2 billion years ago, because their formation is enzyme-independent and emerged in parallel with the biosynthesis of ergosterol and cholesterol [7,10]. In humans and many other vertebrates, vitamin D functions as a hormone that regulates a wide range of endocrine and metabolic processes [16]. By contrast, in plants and fungi vitamin D_2_ is primarily a photochemical end product of UV exposure and does not exert known regulatory functions in these organisms [17].

Vitamin D_2_ can be absorbed by animals and retained through the food chain, providing a dietary source of vitamin D for species possessing the appropriate endocrine machinery [18]. As a consequence, vitamin D accumulates along trophic levels, with higher-order consumers containing greater amounts. This phenomenon is particularly pronounced in sharks, which occupy upper trophic levels and consequently represent some of the richest natural sources of vitamin D in marine ecosystems [19]. Aquatic environments present a special case, as limited penetration of UV-B radiation restricts endogenous vitamin D synthesis; organisms inhabiting deeper waters therefore depend largely on dietary intake. Studies in rainbow trout demonstrate that endogenous vitamin D production is not entirely absent in aquatic species. Vitamin D-deficient animals can synthesize vitamin D in the skin upon exposure to blue light (380–480 nm), via photochemical conversion of 7-dehydrocholesterol driven by visible light rather than UV-B radiation [20]. The resulting vitamin D_3_ is subsequently metabolized to biologically active 1,25(OH)_2_D_3_, which contributes to calcium homeostasis, indicating the presence of a functional vitamin D endocrine system analogous to that of terrestrial vertebrates. The physicochemical mechanisms underlying visible-light-driven vitamin D synthesis in fish skin remain to be elucidated.

Microalgae exhibit a markedly higher capacity for vitamin D production than most higher plants, likely representing an adaptation to variable and often limited UV exposure [21]. Certain microalgal species can produce both vitamin D_2_ and vitamin D_3_, reflecting a complex interplay between sterol composition, photochemistry, and the evolutionary conservation of biosynthetic pathways [22]. Because most microalgae contain higher levels of ergosterol than 7-dehydrocholesterol, vitamin D_2_ is typically the dominant photoproduct [23]. Sterol and vitamin D metabolite concentrations in microalgae vary substantially with species, developmental stage, and UV exposure [22]. Under standard growth conditions, ergosterol and 7-dehydrocholesterol generally account for 0.1–1% of total cellular sterols, but their abundance can increase to approximately 5% under UV stress or altered enzymatic regulation. Vitamin D metabolites themselves usually represent minor, transient photoproducts, accumulating briefly before further metabolism or secretion [24]. The capacity to generate both vitamin D_2_ and vitamin D_3_ may provide selective advantages, potentially including enhanced photoprotection, modulation of membrane properties, and maintenance of sterol-derived photoproducts across fluctuating UV environments [21].

Comparative genomic analyses indicate that CYP710A homologs and other sterol desaturases are highly conserved across green algae and land plants [25], supporting the notion that early divergence of sterol biosynthetic pathways enabled flexibility in vitamin D precursor production.

## 3. Integration of Sterol Biosynthesis and Vitamin D Signaling

Vitamin D metabolites and sterols originate from distinct biochemical precursors and give rise to different end products. Nevertheless, their metabolic pathways and ecological roles intersect throughout evolution and physiology (Figure 1). Circulating and tissue concentrations of vitamin D_2_ and vitamin D_3_ metabolites are generally low [26], despite the large pool of the precursor 7-dehydrocholesterol available for cutaneous vitamin D_3_ synthesis and the additional dietary intake of both vitamin D_2_ and vitamin D_3_ (Table 1).

The low circulating abundance of vitamin D metabolites also reflects their lipophilicity and tightly regulated metabolism and transport. After entering the circulation, vitamin D is rapidly bound to vitamin D-binding protein (DBP), and to a lesser extent to albumin or lipoproteins, with only a minute fraction remaining free [27]. DBP-mediated transport directs vitamin D to the liver and other tissues, while some vitamin D can be sequestered in adipose or other lipid-rich tissues, where concentrations may reach up to approximately 300 nM [28]. Nevertheless, systemic availability is determined largely by binding affinities and metabolic conversion rates rather than by total body stores.

In the liver, CYP2R1 hydroxylates vitamin D_2_ and vitamin D_3_ to form 25(OH)D_2_ and 25(OH)D_3_, which constitute the major circulating vitamin D metabolites [29]. These forms have relatively long half-lives (weeks), making serum 25(OH)D the preferred clinical marker of vitamin D status [30]. Subsequent conversion to the hormonally active metabolites 1,25(OH)_2_D_2_ and 1,25(OH)_2_D_3_ is catalyzed by CYP27B1, predominantly in the kidney but also in some extra-renal tissues [31]. This step is stringently regulated by parathyroid hormone, calcium and phosphate levels, and negative feedback, such that only approximately 0.1–1% of circulating 25(OH)D is converted [32].

Numerous extra-renal tissues and cell types, including immune cells, skin, colon, prostate, breast, and other epithelial tissues, express the *CYP27B1* gene and can locally convert circulating 25(OH)D to 1,25(OH)_2_D_3_ in an autocrine or paracrine manner. Unlike renal calcitriol synthesis, this process is regulated by local cytokines, growth factors, and disease-related signals rather than systemic calcium–phosphate homeostasis, emphasizing its role in tissue-specific functions such as immune regulation, cellular differentiation, and growth control [33]. In cancer, local 1,25(OH)_2_D_3_ production has been linked to anti-proliferative, pro-differentiation, pro-apoptotic, and immunomodulatory effects, indicating that adequate 25(OH)D_3_ availability is essential for these protective mechanisms. Evidence from melanoma and other malignancies suggests that intra-tumoral vitamin D metabolism can influence disease progression and therapeutic response, supporting a role for extra-renal 1,25(OH)_2_D_3_ synthesis in host anti-tumor defense [34]. Dysregulation of this local metabolism may therefore weaken vitamin D-mediated anti-tumor and immunomodulatory effects within the tissue microenvironment, particularly in skin cancer and melanoma.

**Figure 2:**
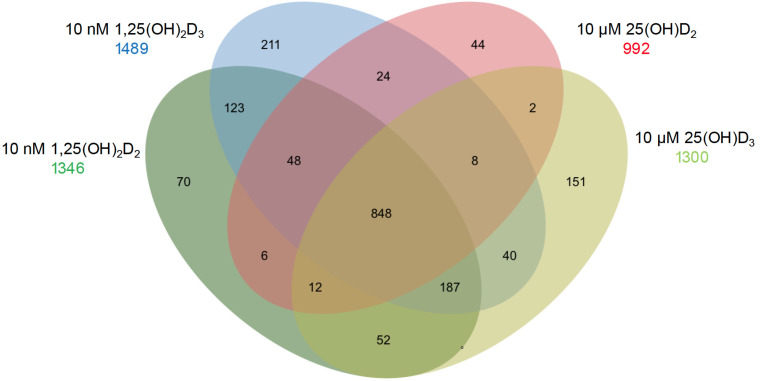
Gene Regulatory Potential of Vitamin D Metabolites. A Venn diagram summarizes RNA sequencing (RNA-seq) results obtained after in vitro stimulation of PBMCs from a healthy individual for 24 h with 10 nM 1,25(OH)_2_D_2_, 10 nM 1,25(OH)_2_D_3_, 10 µM 25(OH)D_2_, or 10 µM 25(OH)D_3_. The numbers indicate significantly regulated vitamin D target genes, defined by a false discovery rate (FDR) < 0.05 [12].

At pharmacological concentrations, the gene-regulatory effects of 1,25(OH)_2_D_2_, 1,25(OH)_2_D_3_, 25(OH)D_2_, and 25(OH)D_3_ were compared in human peripheral blood mononuclear cells (PBMCs) (Figure 2). Transcriptome-wide analyses in this experimental system revealed that approximately 90% of vitamin D target genes regulated by 1,25(OH)_2_D_2_ and 1,25(OH)_2_D_3_ are shared, confirming functional equivalence of the two active metabolites at the transcriptional level.

As described above, UV-B absorption by 7-dehydrocholesterol or ergosterol enables their non-enzymatic conversion into vitamin D_3_ or vitamin D_2_, respectively [1]. This non-enzymatic reaction proceeds via a pre-vitamin D intermediate followed by thermal isomerization. This photochemical sterol-to-vitamin D pathway is not restricted to animals and fungi. Several microalgae, including *Nannochloropsis oceanica*, produce vitamin D_3_ upon UV-B exposure, reaching yields of up to approximately 1 µg/g dry mass [22]. *Emiliania huxleyi* can synthesize both vitamin D_2_ and vitamin D_3_, and vitamin D supplementation or UV exposure has been shown to modulate oxidative stress responses and enhance photosynthetic performance [21]. These observations support the concept that sterol-derived vitamin D production is a deeply conserved biochemical capability across eukaryotes, likely serving primordial functions such as photoprotection, radical scavenging, or membrane stabilization long before the emergence of a dedicated vitamin D endocrine system [35]. Thus, the convergence of sterol biosynthesis, UV-driven photochemistry, and vitamin D formation reflects both ecological adaptation and evolutionary continuity [36]. In animals, this ancient photochemical by-product was subsequently co-opted into a highly regulated endocrine network, generating stable circulating metabolites (25(OH)D) and tightly controlled hormonal effectors (1,25(OH)_2_D).

Concentrations of specific vitamin D metabolites and sterols in healthy individuals exhibit substantial inter-individual variability (Table 1), which can be markedly altered in disease states. Large-scale metabolomics and metabolite quantitative trait locus studies have revealed that genetic variation significantly influences sterol and vitamin D metabolite levels, linking these pathways to disease susceptibility and inter-individual differences in physiological responses [27,37].

## 4. Integrated Enzymatic Networks Linking Vitamin D Activation, Sterol Metabolism, and Tissue-Specific Signaling

Except for SC5D, all sterol biosynthetic enzymes discussed here are oxidoreductases (Figure 1). Following the canonical CYP2R1–CYP27B1 activation pathway described above, tissue-specific regulation of vitamin D signaling is achieved through differential expression of *CYP27B1* and *CYP24A1* [38]. CYP24A1, a vitamin D-responsive 24-hydroxylase, converts 1,25(OH)_2_D_3_ into less active catabolic products, such as 1,24,25(OH)_3_D_3_, thereby maintaining ligand homeostasis. Notably, *CYP24A1* expression in intestinal epithelial cells is minimal under basal conditions but strongly induced by local 1,25(OH)_2_D_3_ [39].

Beyond its role in vitamin D metabolism, CYP27A1 plays a central function in sterol and oxysterol biology by catalyzing the formation of 27-hydroxycholesterol, an endogenous ligand of the nuclear receptors LXRα and LXRβ [40] (Figure 1). Through LXR activation, 27-hydroxycholesterol regulates cholesterol homeostasis, lipid transport, and intestinal epithelial repair. Loss of *CYP27A1* compromises LXR signaling, attenuates epithelial regeneration following injury, and exacerbates intestinal damage [41]. In addition, CYP27A1 contributes to bile acid biosynthesis and shapes cellular oxysterol profiles, thereby intersecting with multiple nuclear receptor pathways, including LXR and pregnane X receptor (PXR) [42].

Comparative insights from plants reveal that sterol biosynthesis shares early steps with animal cholesterol metabolism, beginning with the conversion of acetyl-CoA to mevalonate and subsequently to squalene, but diverges at the level of sterol cyclization [43]. In plants, cycloartenol synthase, or, in some species, lanosterol synthase, converts 2,3-oxidosqualene into the primary sterol scaffold. Subsequent enzymatic modifications, including demethylation, reduction, isomerization, and side-chain alkylation, generate the major plant sterols β-sitosterol, campesterol, and stigmasterol [44]. These reactions are catalyzed by plant-specific sterol-modifying enzymes, such as sterol C14-demethylases and Δ14-reductases, resulting in sterols that are structurally and functionally distinct from cholesterol despite shared biosynthetic principles [45]. Key enzymes of the late steps in plant sterol biosynthesis (Figure 1), including DWF5, DWF1, and the plant ortholog of ERG3, are predominantly localized to the endoplasmic reticulum. However, many of these enzymes also display additional subcellular localizations, such as the plasma membrane or lipid droplets, suggesting a more complex spatial organization of sterol metabolism in plant cells [46].

**Figure 3:**
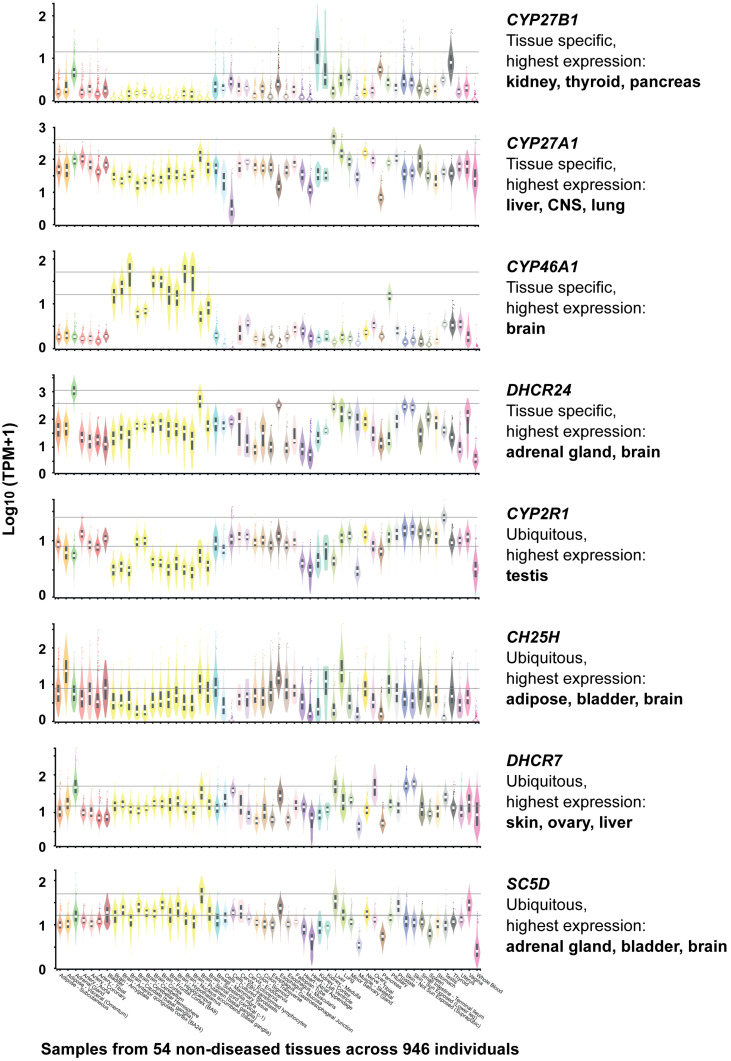
Tissue-Specific expression of Key Genes Involved in Vitamin D and Sterol Metabolism. Based on GTEx data (https://gtexportal.org, accessed on 6 January 2026).

RNA-seq data from the Genotype-Tissue Expression (GTEx) project [47] (https://gtexportal.org, accessed on 6 January 2026) currently provide the most comprehensive resource for comparing human gene expression across tissues. The dataset comprises 54 healthy tissue types obtained from 948 individuals, enabling robust assessment of tissue-specific expression patterns. This large transcriptomic dataset indicates that four enzyme-encoding genes, *CYP27B1*, *CYP27A1*, *CYP46A1*, and *DHCR24*, exhibit pronounced tissue specificity, whereas other pathway components are more ubiquitously expressed (Figure 3).

Although *CYP27B1* is classically associated with renal function, it is also highly expressed in two major endocrine glands, thyroid and pancreas, suggesting a broader endocrine integration of local 1,25(OH)_2_D_3_ production. This is notable, as extra-renal production of 1,25(OH)_2_D_3_ has previously been reported primarily in immune and skin cells [48]. These expression patterns support the concept that vitamin D activation is not exclusively endocrine but may function in tissue-autonomous or paracrine signaling contexts, analogous to oxysterol-mediated LXR activation. Similarly, *CYP27A1*, typically linked to hepatic metabolism, shows elevated expression in the brain. Notably, genes encoding for enzymes involved in cholesterol metabolism and oxysterol production, including *DHCR24*, *CYP46A1*, *CYP27A1*, and *CH25H*, are abundantly expressed in neural tissues, emphasizing the critical role of sterols and their derivatives in brain physiology.

In summary, vitamin D activation is governed by tightly regulated cytochrome P450 enzymes that link sterol biosynthesis, oxysterol signaling, and tissue-specific nuclear receptor activity. These conserved pathways integrate endocrine control, genetic variation, and evolutionary adaptations across kingdoms.

## 5. Evolutionary and Genetic Adaptation of Vitamin D and Sterol Metabolism in Human Populations

Anatomically modern humans emerged approximately 300,000 years ago in East Africa with darkly pigmented skin, an adaptation that provided protection against intense equatorial UV radiation while still allowing sufficient cutaneous synthesis of vitamin D_3_ [49]. During the major dispersal out of Africa around 75,000 years ago, human populations migrated into Asia, Europe, and eventually the Americas, encountering higher latitudes with reduced UV-B availability, colder climates, and increased reliance on clothing [50]. Human skin pigmentation thus reflects an evolutionary trade-off between photoprotection and the requirement for adequate cutaneous vitamin D synthesis. The independent evolution of depigmented skin in European and East Asian populations, together with the emergence of intermediate pigmentation and tanning capacity at mid-latitudes, emphasizes strong selective pressures to maintain vitamin D production across diverse UV environments [50,51]. Collectively, these environmental and behavioral changes substantially increased the risk of vitamin D deficiency.

Despite the persistence of dark skin pigmentation in early European populations for more than 30,000 years, skeletal remains show little evidence of bone pathology attributable to vitamin D deficiency [52]. Over time, populations evolving under different latitudinal and environmental conditions accumulated genetic polymorphisms affecting skin pigmentation, cholesterol and sterol metabolism, vitamin D synthesis, transport, and receptor signaling [53]. These adaptations modulate responses to UV exposure, vitamin D synthesis efficiency, and downstream physiological outcomes, emphasizing the importance of genetic context in interpreting global variation in vitamin D status [54].

Genetic polymorphisms in *CYP2R1*, *CYP27B1*, and *CYP27A1* significantly influence circulating concentrations of 25(OH)D_3_ and 1,25(OH)_2_D_3_, with potential clinical relevance for cardiovascular and other diseases [55,56,57] (Table 2). Particularly strong evidence for adaptive selection is found at the *DHCR7* locus. Regulatory single-nucleotide polymorphisms (SNPs) that reduce *DHCR7* expression increase cutaneous levels of 7-dehydrocholesterol, thereby enhancing vitamin D_3_ synthesis under low-UV conditions [58]. Genome-wide association studies consistently identify *DHCR7* variants as major determinants of vitamin D status, independent of skin pigmentation, highlighting substrate availability as a critical control point [59]. Such variants likely conferred a selective advantage by mitigating the risk of severe vitamin D deficiency during human expansion into higher latitudes with limited UV-B exposure.

Substantial genetic variation is present across sterol and vitamin D metabolic pathways. For example, the *SC5D* gene, which encodes an essential enzyme in the final steps of cholesterol biosynthesis, lacks population-defining variants and instead harbors numerous rare mutations, consistent with strong purifying selection and high evolutionary conservation. In contrast, *DHCR24* is highly conserved at the coding level, with few non-synonymous variants [60]. However, the regulatory SNP rs7551288 has been associated with modest alterations in sterol biosynthesis and lipid-related traits, emphasizing the disproportionate contribution of regulatory variation, rather than common missense changes, to phenotypic modulation of sterol metabolism [61].

More pronounced adaptive signals are observed in regulatory regions of genes controlling vitamin D and oxysterol metabolism. The upstream SNP rs12785878 at the *DHCR7* locus represents one of the strongest genetic determinants of circulating 25(OH)D levels across populations by modulating 7-dehydrocholesterol availability [58]. Similarly, promoter variants in *CH25H* (rs1131706) influence enzyme expression and serum 25-hydroxycholesterol levels, linking sterol metabolism to immune regulation. Additional regulatory variants in *CYP27A1* (rs933994, rs8003602) and intronic variants in *CYP46A1* (rs754203) may affect transcriptional output or tissue-specific cholesterol turnover.

Within the vitamin D activation pathway itself, the *CYP2R1* variant rs10766196 and the variant rs10877012 in *CYP27B1* are repeatedly associated with inter-individual differences in vitamin D status [62], further emphasizing the importance of regulatory variation over coding changes. Collectively, these findings indicate strong conservation of core sterol enzymes alongside adaptive regulatory variation in their expression.

Taken together, human migration across latitudes drove regulatory genetic adaptations in vitamin D and sterol pathways, optimizing UV-dependent synthesis, substrate availability, and immune–metabolic responses under diverse environmental pressures.

## 6. Ligand Specificity, Structural Determinants, and Dietary Modulation of VDR and LXR Signaling

VDR binds its natural ligand 1,25(OH)_2_D_3_ with exceptionally high affinity in the sub-nanomolar range (approximately 0.1–0.3 nM) [63]. Structural studies reveal that 1,25(OH)_2_D_3_ occupies a well-defined pocket within the VDR ligand-binding domain (LBD), where binding is stabilized by a precise network of hydrogen bonds between the ligand’s three hydroxyl groups and specific amino acid residues of the receptor [64]. The orientation of the A-ring and the conformation of the flexible side chain further optimize these interactions and contribute to the remarkable stability of the ligand–receptor complex. All three hydroxyl groups, 1-OH and 3-OH on the A-ring and 25-OH on the side chain, are essential for high-affinity binding; modification or removal of any of these moieties markedly reduces affinity and often alters biological activity [65].

In contrast, LXRα and LXRβ bind their endogenous oxysterol ligands with moderate affinity, typically in the low- to mid-nanomolar range, depending on the metabolite [66]. Among the most potent physiological LXR agonists is 27-hydroxycholesterol, generated by CYP27A1 as discussed above. Crystal-structure analyses show that oxysterols bind deeply within the hydrophobic LXR LBD, where the sterol nucleus engages in extensive van der Waals interactions, while hydroxyl groups at the 24, 25, or 27 positions form key hydrogen bonds with polar residues that anchor the ligand [67]. Correct positioning of the sterol side chain and precise orientation of the hydroxyl substituent are critical determinants of binding strength and transcriptional potency; alterations to either the hydroxyl group or the sterol framework substantially weaken receptor activation. Consistent with their distinct ligand-binding properties, dietary sterols differentially modulate VDR and LXR signaling.

Dietary patterns influence nuclear receptor signaling by shaping the spectrum and concentration of available ligands [68]. Omnivorous diets typically provide higher amounts of cholesterol (approximately 350–400 mg/day), leading to increased generation of cholesterol-derived oxysterols that act as potent LXR agonists regulating cholesterol efflux, lipid metabolism, and inflammatory responses. In contrast, vegetarian and vegan diets are enriched in plant sterols (approximately 200–500 mg/day from nuts, seeds, legumes, and fortified foods), which can undergo oxidation during food processing or in vivo to form oxyphytosterols [69]. These oxidized phytosterols are detectable in human plasma and may weakly activate LXR in specific cellular contexts, particularly in tissues directly exposed to dietary sterols [70]. While cholesterol-derived oxysterols are well-established endogenous LXR ligands with genome-wide transcriptional effects, the role of oxidized phytosterols as physiologically relevant LXR agonists remains incompletely defined. However, genome-wide analyses of LXR binding and transcriptional responses to oxyphytosterols remain limited, leaving their physiological relevance incompletely resolved.

A parallel principle applies to VDR signaling. Omnivores primarily obtain vitamin D_3_ from animal-derived foods or supplements, whereas vegetarians and vegans rely more heavily on vitamin D_2_ from plant or fungal sources, or on algal-derived vitamin D_3_ supplements [71]. Both forms support effective VDR activation, resulting in broadly comparable transcriptional responses despite differences in precursor origin and pharmacokinetics.

Together, these examples illustrate how diet shapes nuclear receptor signaling not merely through nutrient quantity but through ligand diversity. Distinct molecular inputs, cholesterol versus phytosterols for LXR, vitamin D_3_ versus vitamin D_2_ for VDR, can converge on similar receptor activation profiles, ultimately influencing lipid metabolism, immune regulation, and metabolic health.

## 7. Conclusions

The evolution of sterols and vitamin D reflects deep biochemical continuity across life, extending from UV-driven photochemical processes in plants, fungi, and algae to tightly regulated endocrine signaling systems in humans. Sterol precursors such as 7-dehydrocholesterol and ergosterol initially shaped membrane architecture and conferred photoprotective advantages in early eukaryotes, while simultaneously enabling the non-enzymatic formation of vitamin D metabolites. Over evolutionary time, these photochemical by-products were co-opted into hormonally active pathways governed by precise enzymatic and receptor-mediated control.

Genetic polymorphisms affecting vitamin D-activating enzymes, as well as transport and receptor components, modulate vitamin D synthesis, sterol metabolism, and signaling efficiency. Evolutionary pressures, including latitude-dependent UV exposure, dietary availability, and environmental constraints, have left discernible signatures in these pathways, accounting for substantial inter-individual and population-level variability in vitamin D status, sterol homeostasis, and associated physiological outcomes.

Dietary patterns further exemplify the functional flexibility of these systems. Omnivorous diets predominantly activate VDR and LXR through cholesterol-derived oxysterols and vitamin D_3_, whereas vegetarian and vegan diets rely more heavily on oxyphytosterols and vitamin D_2_, respectively. Thus, distinct dietary sterol and vitamin D sources converge on shared nuclear receptor signaling programs that support cholesterol homeostasis, metabolic regulation, and immune function. Future work integrating longitudinal metabolomics, tissue-resolved transcriptomics, and genotype-informed intervention studies will be essential to determine how this sterol–vitamin D network can be harnessed for personalized nutritional and therapeutic strategies.

## Figures and Tables

**Table 1:** Concentration of Selected Vitamin D Metabolites and Sterols in Healthy Humans, Fungi and Higher Plants. Reported concentrations are derived from publicly available online databases and refer to physiological conditions in healthy organisms. Human values correspond to circulating levels measured in the blood of healthy individuals, whereas plant and fungal values represent concentrations in fresh tissues of healthy specimens. Data were compiled from CropMetabolome Database (www.cropmetabolome.com), Human Metabolome Database (www.hmdb.ca), and RefMetaDB (www.biosino.org/RefMetaDB); all accessed during November and December 2025.

Metabolite	In Human Plasma (nM)		In Fungi (nM)		In Higher Plants (nM)	
	min.	max.	min.	max.	min.	max.
lathosterol	2380	9900	0	0	0	0
desmosterol	790	2900	0	0	0	0
7-dehydrocholesterol	2800	7200	0	0	1	50
cholesterol	1,000,000	6,500,000	300	40,000	100	30,000
ergosterol	9.3	14.9	150,000	1,200,000	13	6300
episterol	0	0	250	62,700	25	2500
5-dehydroepisterol	0	0	25	25,200	2	1260
campesterol	240	87,000	0.2	10	25	500
brassicasterol	250	1800	0.1	5	10	200
vitamin D_3_	1	5	0	0	20	2600
vitamin D_2_	1	3	2520	380,000	25	12,600
25(OH)D_3_	50	125	0	0	0	0
25(OH)D_2_	2	30	0	0	0	0
1,25(OH)_2_D_3_	0.03	0.16	0	0	0	0
1,25(OH)_2_D_2_	0.001	0.02	0	0	0	0
25-hydroxycholesterol	25	200	0	50	0	30
27-hydroxycholesterol	13	538	0	50	0	30
24-hydroxycholesterol	120	250	0	50	0	30
7-ketocampesterol	1.5	3.0	0.25	627	10	500
7β-hydroxycampesterol	0.8	5.0	0.25	620	25	100
7β-hydroxybrassicasterol	0.6	1.0	3.0	15	5	60

**Table 2:** SNP Locations and Population-Specific Variation in Key Enzymes of Vitamin D and Sterol Metabolic Pathways. SNP data were compiled from the 1000 Genomes Project (www.internationalgenome.org, accessed on 6 January 2026) and Ensembl (www.ensembl.org/index.html, accessed on 6 January 2026) databases. Nucleotide variants are color-coded as follows: guanine (G), orange; adenine (A), green; cytosine (C), blue; thymine (T), red. Population groups are defined as: AFR, African; AMR, Admixed American; EAS, East Asian; EUR, European; SAS, South Asian; TSS, transcription start site.

Gene	SNP	Location	Genetic Variation in Populations
*DHCR7*	rs12785878	Regulatory, 3.53 kb upstream of TSS	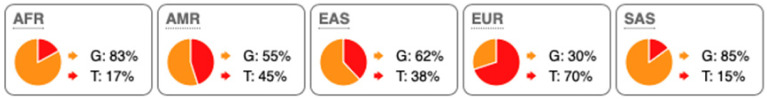
*CYP27B1*	rs10877012	Regulatory, 1.26 kb upstream of TSS	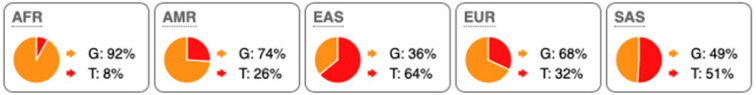
*CYP27A1*	rs933994	Regulatory, 3.75 kb downstream of TSS	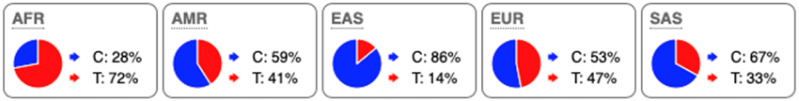
*CYP46A1*	rs8003602	Regulatory, 1.79 kb upstream of TSS	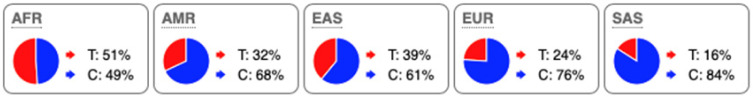
*CYP46A1*	rs754203	Regulatory, 4.46 kb downstream of TSS	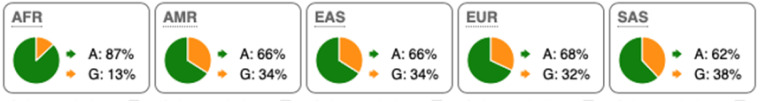
*CYP2R1*	rs10766196	Regulatory, 1.46 kb upstream of TSS	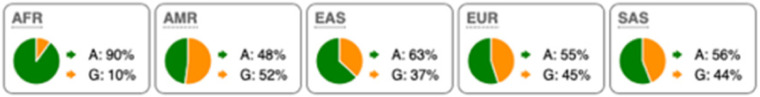
*DHCR24*	rs7551288	Regulatory, 12.3 kb downstream of TSS	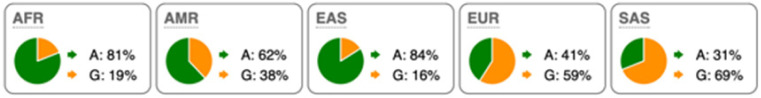
*CH25H*	rs1131706	Regulatory, 6.60 kb upstream of TSS	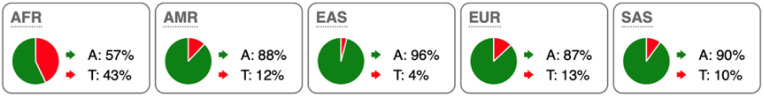

## Data Availability

No new data were created or analyzed in this study.
